# Supplementary material for: Regulation by cyclic di-GMP attenuates dynamics and enhances robustness of bimodal curli gene activation in Escherichia coli
Source: PLoS Genet. 2023 May 15;19(5):e1010750. doi: 10.1371/journal.pgen.1010750 (PMC10212085; doi:10.1371/journal.pgen.1010750)
Supplement: S1 Table — (PDF) [file pgen.1010750.s001.pdf]

S1 Table. ***E. coli*** **strains and plasmids used in this study.**

Strains	Relevant genotype	Reference
W3110	W3110 derivative with functional RpoS	[1]
VS1146	W3110 *csgA*::*csgA*_RBS_*sfgfp*	[2]
VS1857	VS1146 Δ*mlrA*	This work
VS1732	VS1146 Δ*pdeH*	This work
VS1720	VS1146 Δ*dgcE*	This work
VS1258	VS1146 Δ*pdeR*	This work
VS1257	VS1146 Δ*dgcM*	This work
VS1717	VS1146 Δ*pdeH* Δ*dgcE*	This work
VS1713	VS1146 Δ*pdeR* Δ*dgcM*	This work
VS1885	VS1146 Δ*pdeH* Δ*dgcE* Δ*pdeR*	This work
VS1729	VS1146 Δ*pdeH* Δ*dgcE* Δ*pdeR* Δ*dgcM*	This work
Plasmids		
pTrc99a	Expression vector; *Ptrc* promoter inducible by isopropyl-β-D-thiogalactopyranoside (IPTG); pBR ori; Ap^R	[3]
pVS2689	pTrc99a::*pdeH*	This work
pVS1644	pTrc99a::*dgcE*	This work

References
1. Serra DO, Richter AM, Klauck G, Mika F, Hengge R. Microanatomy at cellular resolution and spatial order of physiological differentiation in a bacterial biofilm. mBio. 2013;4: e00103-13. PMID: 23512962.
2. Besharova O, Suchanek VM, Hartmann R, Drescher K, Sourjik V. Diversification of gene expression during formation of static submerged biofilms by *Escherichia coli*. Front Microbiol. 2016;7: 1568. PMID: 27761132.
3. Amann E, Ochs B, Abel K-J. Tightly regulated tac promoter vectors useful for the expression of unfused and fused proteins in *Escherichia coli*. Gene. 1988;69: 301-15. PMID: 3069586.
