# Supplementary material for: Regulation by cyclic di-GMP attenuates dynamics and enhances robustness of bimodal curli gene activation in Escherichia coli
Source: PLoS Genet. 2023 May 15;19(5):e1010750. doi: 10.1371/journal.pgen.1010750 (PMC10212085; doi:10.1371/journal.pgen.1010750)
Supplement: S1 Fig — Wild-type E. coli cultures were grown as in Fig 1A (except shaking conditions) but with different indicated concentrations of TB. (A) Bacterial growth and activity of transcriptional curli reporter. Error bars indicate SEM of 6 technical replicates. (B) Distribution of single-cell fluorescence levels after 24 h of growth in a plate reader measured by flow cytometry. Note that the scale in the y axes is different for individual conditions to improve readability. (PDF) [file pgen.1010750.s002.pdf]

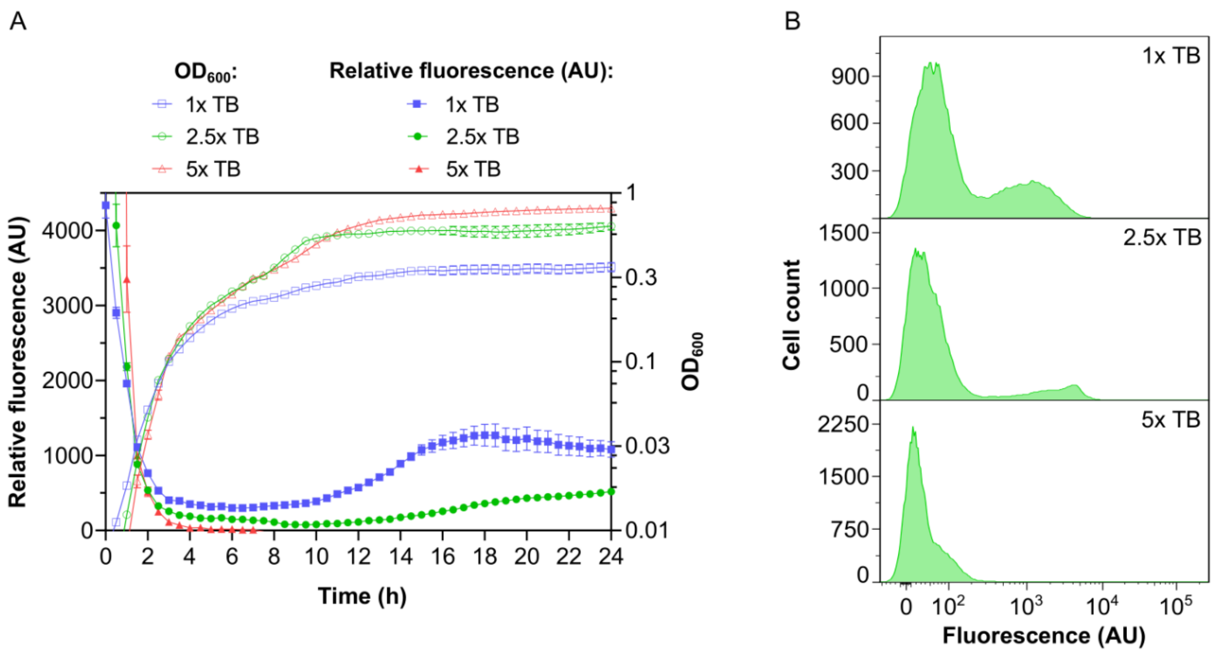

S1 Fig. Dependence of curli gene expression on nutrient levels. Wild-type *E. coli* cultures were grown as in Fig 1A (except shaking conditions) but with different indicated concentrations of TB. **(A)** Bacterial growth and activity of transcriptional curli reporter. Error bars indicate SEM of 6 technical replicates. **(B)** Distribution of single-cell fluorescence levels after 24 h of growth in a plate reader measured by flow cytometry. Note that the scale in the y axes is different for individual conditions to improve readability.
