# Supplementary material for: Regulation by cyclic di-GMP attenuates dynamics and enhances robustness of bimodal curli gene activation in Escherichia coli
Source: PLoS Genet. 2023 May 15;19(5):e1010750. doi: 10.1371/journal.pgen.1010750 (PMC10212085; doi:10.1371/journal.pgen.1010750)
Supplement: S2 Fig — Wild-type E. coli cultures were grown as in Fig 1A (except shaking conditions) but with addition of either indicated concentrations of serine hydroxamate (SHX) at inoculation point or 1 mM serine after 6 h of growth. (A) Bacterial growth and activity of transcriptional curli reporter. Error bars indicate SEM of 6 technical replicates. (B) Distribution of single-cell fluorescence levels after 24 h of growth in a plate reader measured by flow cytometry. (PDF) [file pgen.1010750.s003.pdf]

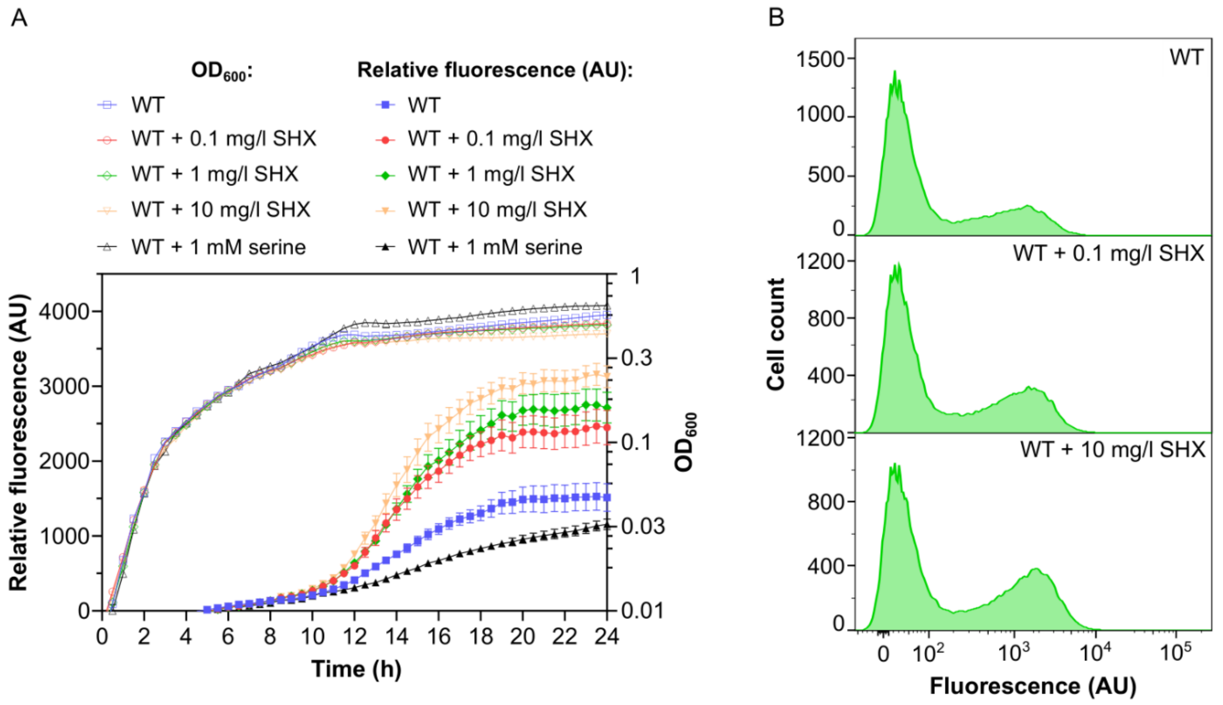

S2 Fig. Stimulation of curli gene expression by stringent response. Wild-type *E. coli* cultures were grown as in Fig 1A (except shaking conditions) but with addition of either indicated concentrations of serine hydroxamate (SHX) at inoculation point or 1 mM serine after 6 h of growth. **(A)** Bacterial growth and activity of transcriptional curli reporter. Error bars indicate SEM of 6 technical replicates. **(B)** Distribution of single-cell fluorescence levels after 24 h of growth in a plate reader measured by flow cytometry.
