# Supplementary material for: Regulation by cyclic di-GMP attenuates dynamics and enhances robustness of bimodal curli gene activation in Escherichia coli
Source: PLoS Genet. 2023 May 15;19(5):e1010750. doi: 10.1371/journal.pgen.1010750 (PMC10212085; doi:10.1371/journal.pgen.1010750)
Supplement: S3 Fig — Error bars indicate SEM of 5 technical replicates. E. coli planktonic cultures of the wild-type (WT), ΔmlrA strain, and individual, double and quadruple deletions of DGC or PDE enzymes were grown in TB in a plate reader as in Fig 1A (except shaking conditions). (PDF) [file pgen.1010750.s004.pdf]

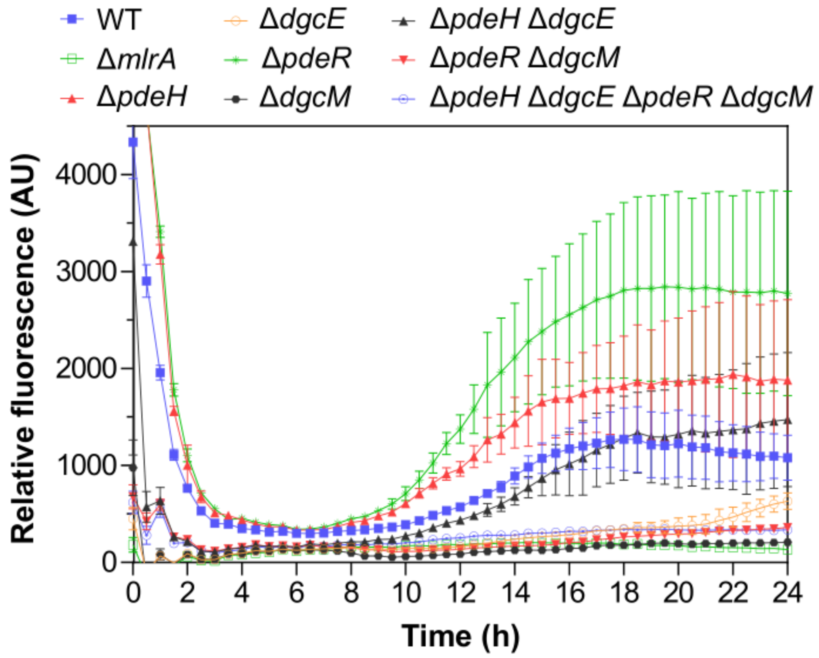

S3 Fig. Relative fluorescence of curli reporter in planktonic culture grown in TB medium in a plate reader. Error bars indicate SEM of 5 technical replicates. *E. coli* planktonic cultures of the wild-type (WT), Δ*mlrA* strain, and individual, double and quadruple deletions of DGC or PDE enzymes were grown in TB in a plate reader as in Fig 1A (except shaking conditions)
