# Supplementary material for: Regulation by cyclic di-GMP attenuates dynamics and enhances robustness of bimodal curli gene activation in Escherichia coli
Source: PLoS Genet. 2023 May 15;19(5):e1010750. doi: 10.1371/journal.pgen.1010750 (PMC10212085; doi:10.1371/journal.pgen.1010750)
Supplement: S4 Fig — Wild-type (WT) and ΔpdeH ΔdgcE ΔpdeR ΔdgcM strains were grown in TB in a plate reader as in S3 Fig, but during 36 h. (A) Induction of transcriptional curli reporter. Error bars indicate SEM of 10 technical replicates. (B) Distribution of single-cell fluorescence levels in populations of both strains after 36 h of growth measured by flow cytometry. (PDF) [file pgen.1010750.s005.pdf]

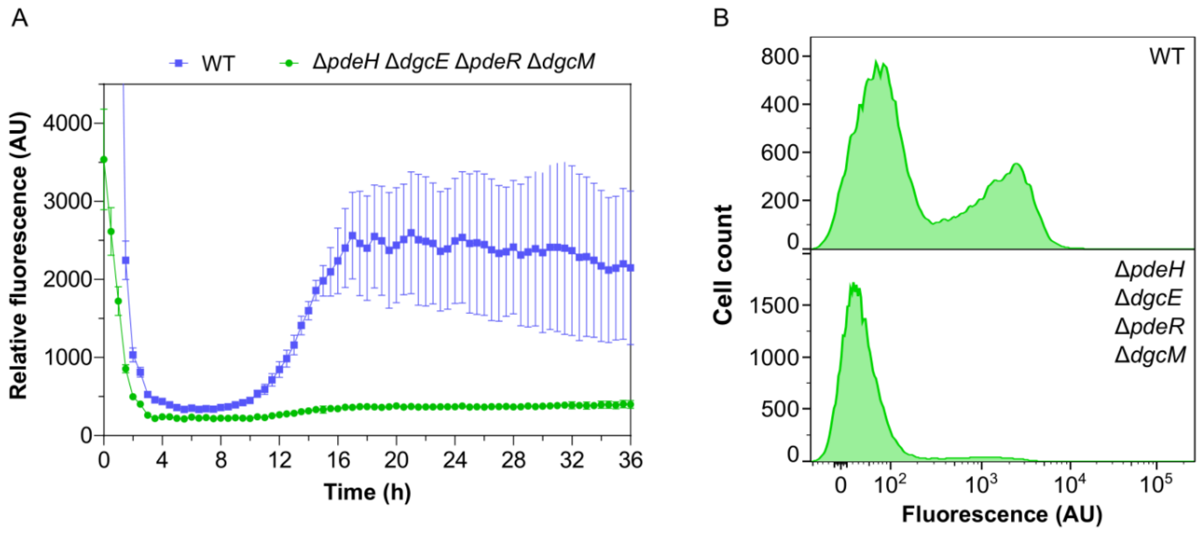

S4 Fig. Curli gene expression upon prolonged cultivation in a plate reader. Wild-type (WT) and Δ*pdeH* Δ*dgcE* Δ*pdeR* Δ*dgcM* strains were grown in TB in a plate reader as in S3 Fig, but during 36 h. **(A)** Induction of transcriptional curli reporter. Error bars indicate SEM of 10 technical replicates. **(B)** Distribution of single-cell fluorescence levels in populations of both strains after 36 h of growth measured by flow cytometry.
