# Supplementary material for: Regulation by cyclic di-GMP attenuates dynamics and enhances robustness of bimodal curli gene activation in Escherichia coli
Source: PLoS Genet. 2023 May 15;19(5):e1010750. doi: 10.1371/journal.pgen.1010750 (PMC10212085; doi:10.1371/journal.pgen.1010750)
Supplement: S5 Fig — Biofilm formation by indicated strains grown in multi-well plates in TB medium, quantified using crystal violet (CV) staining. Error bars indicate SEM of 3 independent replicates. (PDF) [file pgen.1010750.s006.pdf]

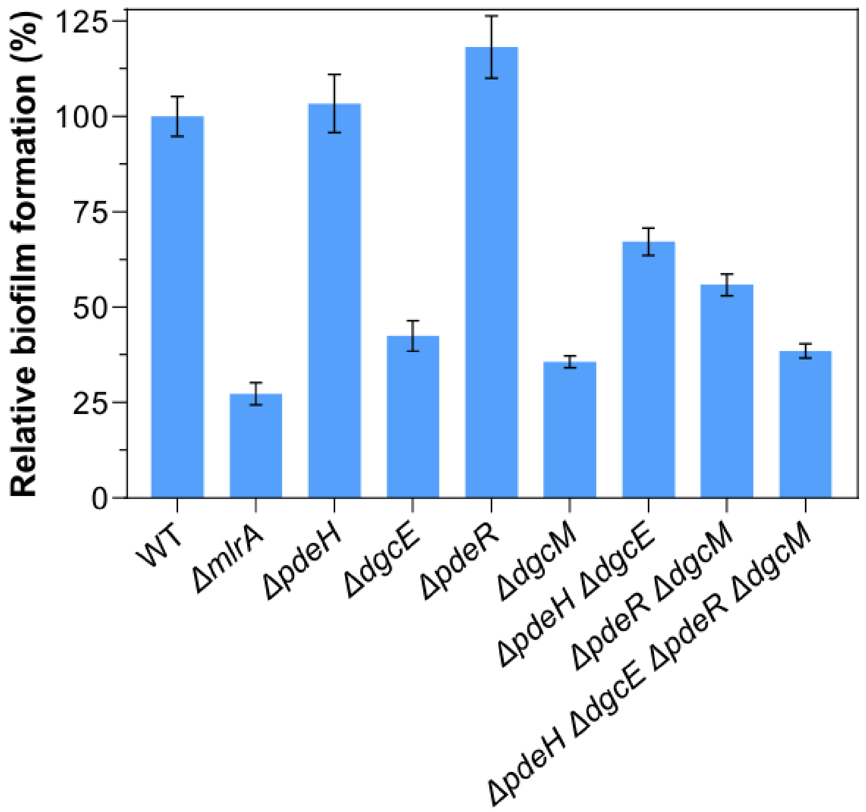

S5 Fig. Curli gene expression in submerged biofilm cultures. Biofilm formation by indicated strains grown in multi-well plates in TB medium, quantified using crystal violet (CV) staining. Error bars indicate SEM of 3 independent replicates.
