# Supplementary material for: Regulation by cyclic di-GMP attenuates dynamics and enhances robustness of bimodal curli gene activation in Escherichia coli
Source: PLoS Genet. 2023 May 15;19(5):e1010750. doi: 10.1371/journal.pgen.1010750 (PMC10212085; doi:10.1371/journal.pgen.1010750)
Supplement: S6 Fig — Images of macrocolonies of indicated strains after 8 days of growth on salt-free LB agar plates. (PDF) [file pgen.1010750.s007.pdf]

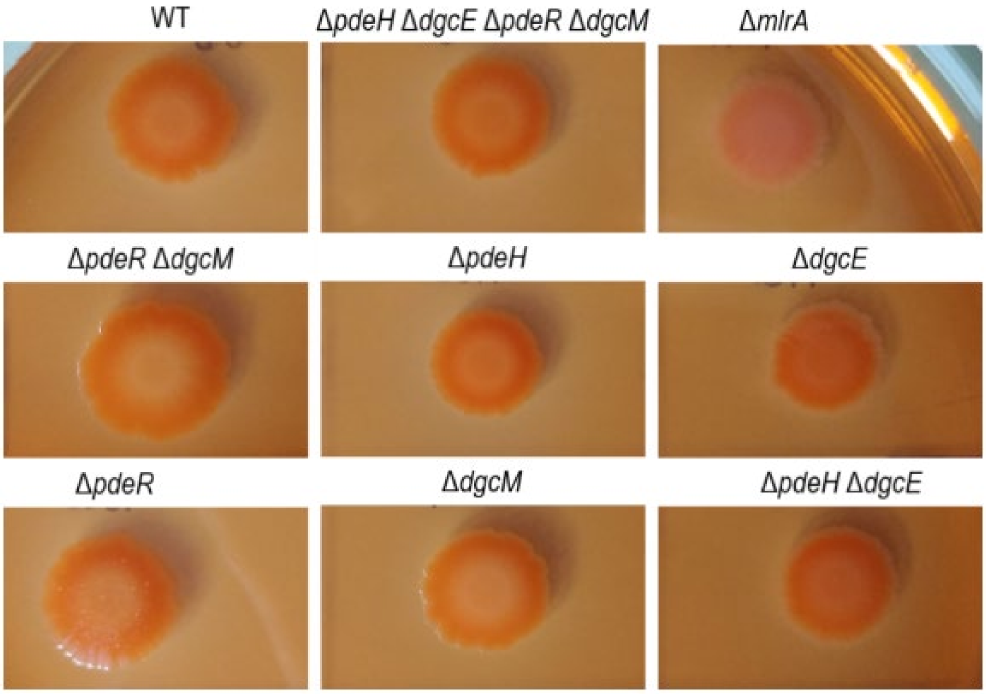

S6 Fig. Curli expression in macrocolony biofilms. Images of macrocolonies of indicated strains after 8 days of growth on salt-free LB agar plates.
