# Supplementary material for: Regulation by cyclic di-GMP attenuates dynamics and enhances robustness of bimodal curli gene activation in Escherichia coli
Source: PLoS Genet. 2023 May 15;19(5):e1010750. doi: 10.1371/journal.pgen.1010750 (PMC10212085; doi:10.1371/journal.pgen.1010750)
Supplement: S7 Fig — Shown are kernel density estimates of curli expression in the wild-type WT, individual and quadruple deletions of DGC or PDE enzymes at selected time points. Cells in stationary phase were loaded into mother machine chip supplied with fresh medium, and then switched to the conditioned media at the “0 h” time point. Note that the scale in the y axes is different for individual conditions to improve readability. (PDF) [file pgen.1010750.s008.pdf]

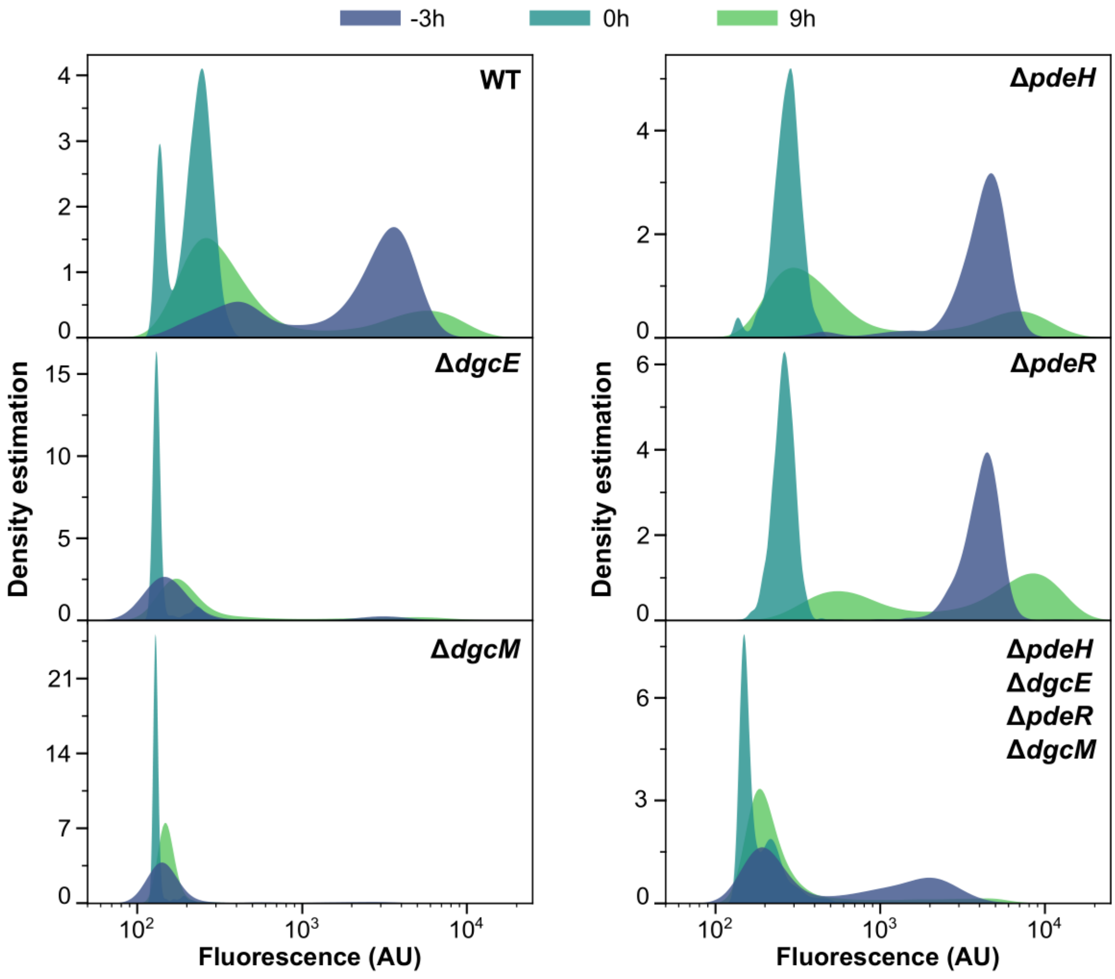

S7 Fig. Distributions of curli expression at different time points in the microfluidics experiment. Shown are kernel density estimates of curli expression in the wild-type WT, individual and quadruple deletions of DGC or PDE enzymes at selected time points. Cells in stationary phase were loaded into mother machine chip supplied with fresh medium, and then switched to the conditioned media at the ‘’0 h’’ time point. Note that the scale in the y axes is different for individual conditions to improve readability.
