# Supplementary material for: Regulation by cyclic di-GMP attenuates dynamics and enhances robustness of bimodal curli gene activation in Escherichia coli
Source: PLoS Genet. 2023 May 15;19(5):e1010750. doi: 10.1371/journal.pgen.1010750 (PMC10212085; doi:10.1371/journal.pgen.1010750)
Supplement: S8 Fig — Stationary phase cells were introduced into mother machine devices, supplied with fresh medium and then switched to conditioned medium after 4 h of growth, as in Fig 6. (A) Median instantaneous growth rates for the wild-type and for the ΔpdeH ΔdgcE ΔpdeR ΔdgcM strain disabled in c-di-GMP regulation. Growth rate drops rapidly and cells switch on curli expression after a switch to conditioned medium. Shaded area is interquartile range. (B) Fraction of cells with fluorescence exceeding 1000 units. (C) Number of detected cells. Two biological replicates (r1 and r2) were performed for each strain; data for the r1 replicate are also shown in Fig 6. Note that in the r2 experiment for the quadruple deletion strain, cells were only imaged after medium switching. (PDF) [file pgen.1010750.s009.pdf]

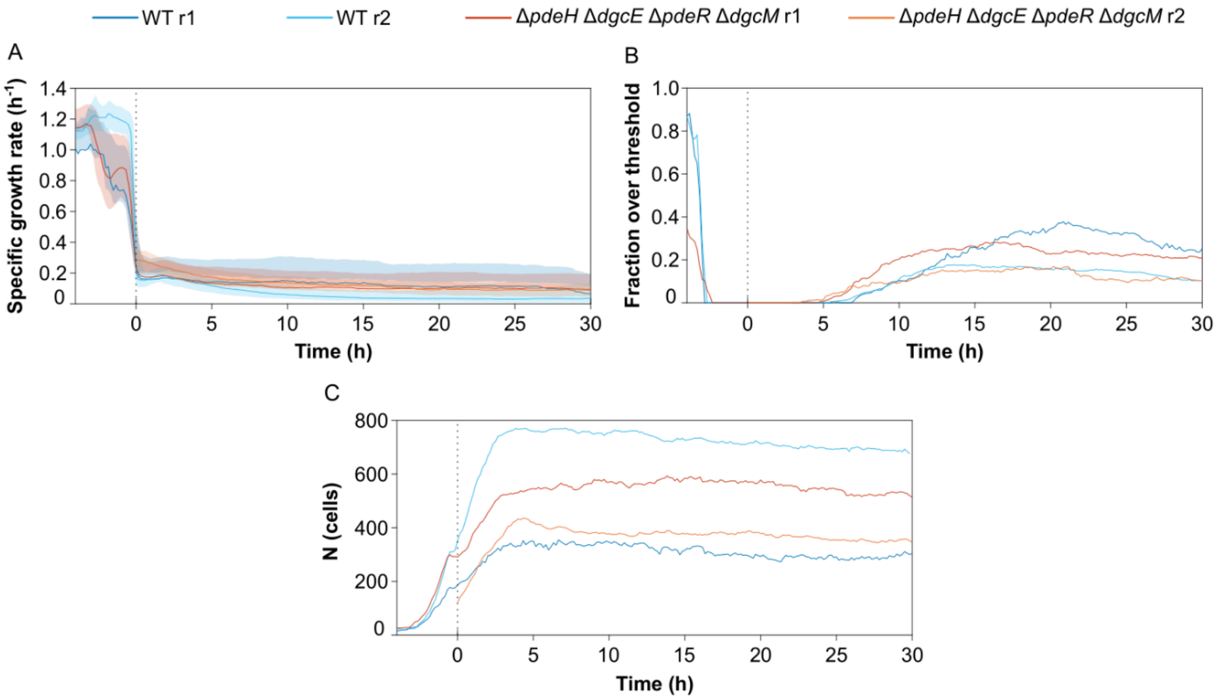

S8 Fig. Growth rates and fraction of curli expressing cells over time. Stationary phase cells were introduced into mother machine devices, supplied with fresh medium and then switched to conditioned medium after 4 h of growth, as in Fig 6. **(A)** Median instantaneous growth rates for the wild-type and for the Δ*pdeH* Δ*dgcE* Δ*pdeR* Δ*dgcM* strain disabled in c-di-GMP regulation. Growth rate drops rapidly and cells switch on curli expression after a switch to conditioned medium. Shaded area is interquartile range. **(B)** Fraction of cells with fluorescence exceeding 1000 units. **(C)** Number of detected cells. Two biological replicates (r1 and r2) were performed for each strain; data for the r1 replicate are also shown in Figure 6. Note that in the r2 experiment for the quadruple deletion strain, cells were only imaged after medium switching.
