# Supplementary material for: Regulation by cyclic di-GMP attenuates dynamics and enhances robustness of bimodal curli gene activation in Escherichia coli
Source: PLoS Genet. 2023 May 15;19(5):e1010750. doi: 10.1371/journal.pgen.1010750 (PMC10212085; doi:10.1371/journal.pgen.1010750)
Supplement: S9 Fig — Data are from the same biological replicates (r1 and r2) as in S8 Fig. (PDF) [file pgen.1010750.s010.pdf]

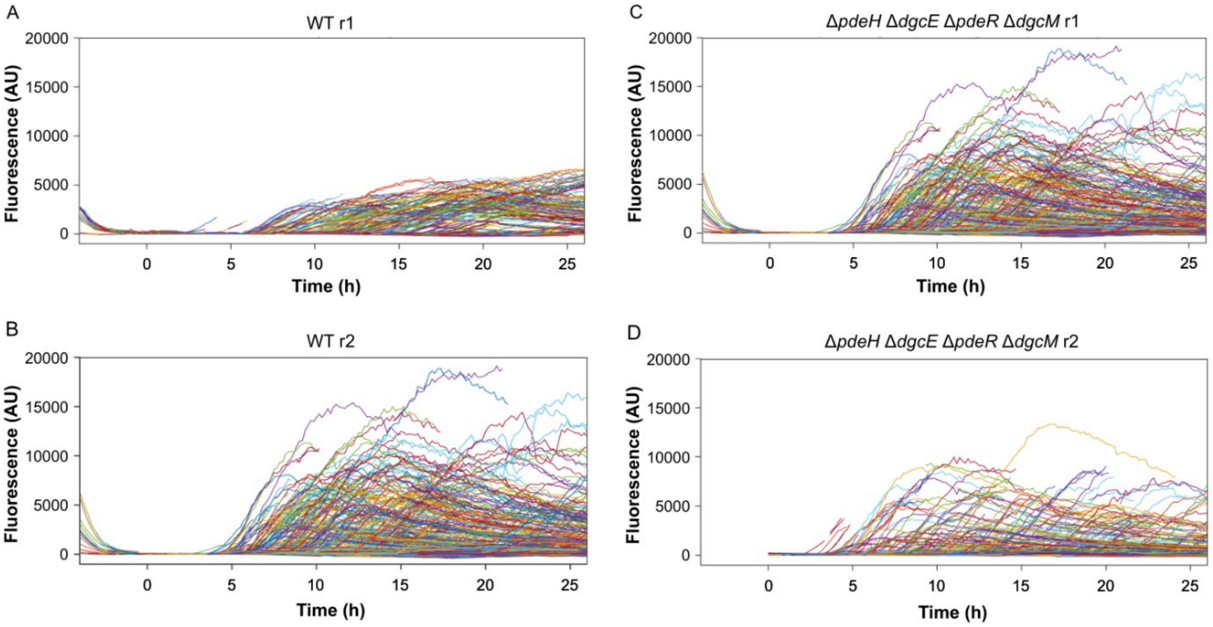

S9 Fig. Single-cell traces of cell fluorescence for all cells for the wild-type and for the c-di-GMP-regulation disabled strain. Data are from the same biological replicates (r1 and r2) as in S8 Fig.
