# Supplementary material for: Regulation by cyclic di-GMP attenuates dynamics and enhances robustness of bimodal curli gene activation in Escherichia coli
Source: PLoS Genet. 2023 May 15;19(5):e1010750. doi: 10.1371/journal.pgen.1010750 (PMC10212085; doi:10.1371/journal.pgen.1010750)
Supplement: S10 Fig — (A) Median curli expression, (B) production rate, and (C) number of cells for traces from both microfluidics experiments (r1 and r2) aligned by the time at which they exceeded a threshold of 103 fluorescence units. Shaded area is interquartile range. Compared to the WT, the rate of curli induction is faster but traces show more variability in a mutant without the global or local c-di-GMP regulatory modules. (PDF) [file pgen.1010750.s011.pdf]

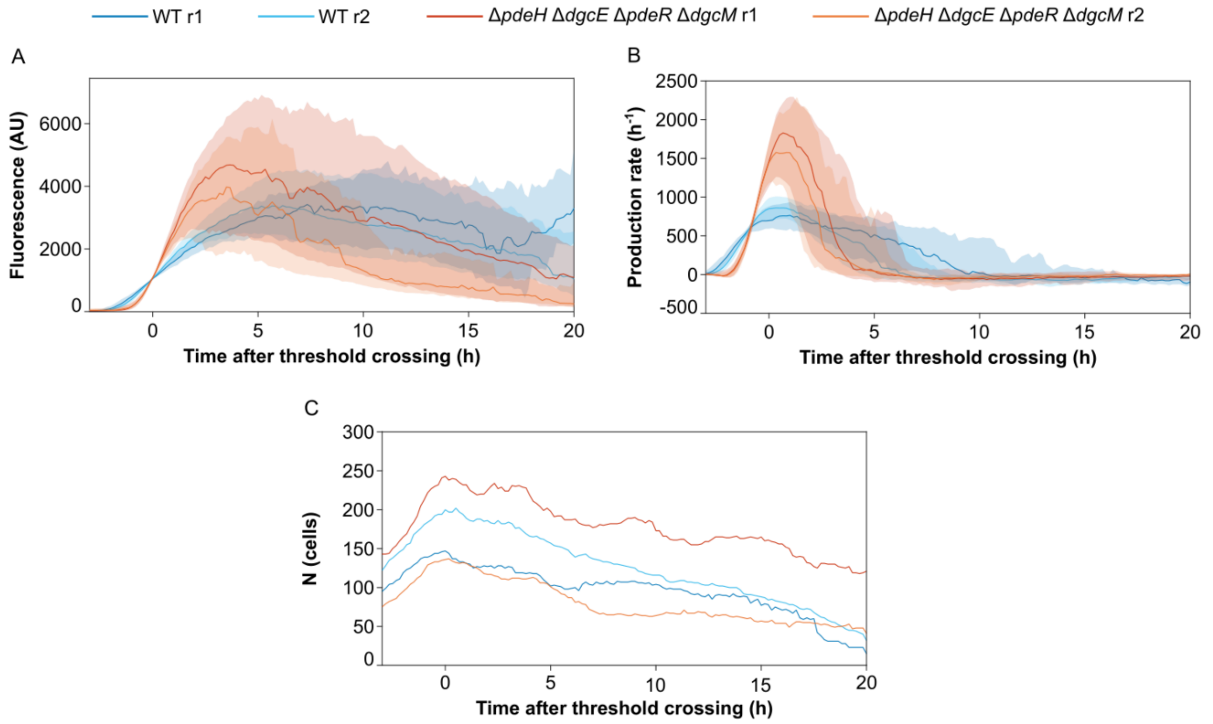

S10 Fig. The rate and variability of curli induction for the wild-type and for the c-di-GMP-regulation disabled strain. (A) Median curli expression, **(B)** production rate, and **(C)** number of cells for traces from both microfluidics experiments (r1 and r2) aligned by the time at which they exceeded a threshold of 103 fluorescence units. Shaded area is interquartile range. Compared to the WT, the rate of curli induction is faster but traces show more variability in a mutant without the global or local c-di-GMP regulatory modules.
