# Supplementary material for: Regulation by cyclic di-GMP attenuates dynamics and enhances robustness of bimodal curli gene activation in Escherichia coli
Source: PLoS Genet. 2023 May 15;19(5):e1010750. doi: 10.1371/journal.pgen.1010750 (PMC10212085; doi:10.1371/journal.pgen.1010750)
Supplement: S1 Protocol — (PDF) [file pgen.1010750.s012.pdf]

S1 Protocol. Design and fabrication of the mother machine.

A microfluidic device was used for time-resolved studies of *E. coli* growth and curli expression, which features mother machines traps for the observation of one-dimensional cell growth. The design of the microfluidic device is shown in Fig 5. The channel structure of the microfluidic chip features four independent channels with a depth of 8 µm, allowing the realization of four conditions, e.g. different media compositions, in one experiment. Each channel contains two inlets enabling on-chip switching between two different media, which are supplied at defined pressures. By controlling the pressure ratio between the two inlets, the medium supplied at the higher pressure flows through the central channel following the junction, while the other medium is pushed away from the junction and flows out through the waste channel (Fig 5D). The medium which flows through the central channel reaches the mother machine cultivation sites before exiting the chip through a single outlet.

The device was produced using a two-layer soft lithography method as described previously. Based on our in-house made design of the channel layout, a 100 mm silicon wafer was produced by e-beam lithography (ConScience, Sweden). The wafer contains the channel layout as a positive relief. The mother machine traps, which are shown in blue in Fig 5A-C, were structured by etching the wafer by 0.8 µm, giving the mother machine the appropriate vertical dimension for cell trapping. The supply channels, which are shown in green in Fig 5A-C, are implemented as photoresist structures with a height of 8 µm on the wafer. The wafer served as a master mold for liquid polydimethylsiloxane (Sylgard 184 PDMS, VwR International GmbH, Germany), which was mixed at volumetric ratio of 7:1 with a cross-linking agent, degassed in a desiccator for 30 min and poured over the wafer to a height of approximately 4 mm and thermally cured at 80 °C overnight. The cured PDMS was peeled off from the wafer and manually cut into separate chips. Inlet and outlet holes were punched with a 0.75 mm punching tool (Robbins True-Cut Disposable Biopsy Punch 0.75mm with Plunger, Robbins Instruments, USA). The surface of the chip was cleaned by a rinse with isopropanol and the application of adhesive tape (tesafilm, Germany) prior to bonding. The chip was irreversibly bonded to a glass substrate by applying oxygen plasma to both chip and glass surfaces (Diener Femto, Diener GmbH, Germany) and bringing the treated surfaces together. The bond was strengthened by storing the bonded device in the oven at 80 °C for 2 min. The device was mounted on an inverted fluorescence microscope (Nikon Eclipse Ti, Nikon Corporation, Japan) equipped with an incubator. The microscope setup included an Andor Zyla 4.2 sCMOS camera (Oxford Instruments, UK), an objective with 100x magnification (Plan Apochromat λ Oil, NA=1.45, WD=170 µm; Nikon, Japan) and a perfect focus system (Nikon Corporation, Japan) for focus drift compensation.
