# Supplementary material for: Regulation by cyclic di-GMP attenuates dynamics and enhances robustness of bimodal curli gene activation in Escherichia coli
Source: PLoS Genet. 2023 May 15;19(5):e1010750. doi: 10.1371/journal.pgen.1010750 (PMC10212085; doi:10.1371/journal.pgen.1010750)
Supplement: S2 Protocol — (PDF) [file pgen.1010750.s013.pdf]

S2 Protocol. Growth experiments in the microfluidics device.

E. coli cells were allowed to grow in TB medium until the stationary phase prior to inoculation into the device. Conditioned medium was prepared by cultivating the wild-type *E. coli* cells in TB medium for 20 h (batch culture), after which the cell suspension was centrifuged at 4000 rpm for 10 min. The medium then was filter sterilized and stored at 4°C. The mother machine growth sites were loaded with the undiluted cell suspension by manual infusion of the cell suspension through one of the two inlets using a 1-ml syringe. The connection between the syringe and the chip was realized by tygon tubing (Tygon S-54-HL, inner diameter = 0.51 mm, outer diameter = 1.52 mm, VwR International GmbH, Germany) in combination with blunt dispensing needles (general purpose tips, inner diameter = 0.41 mm, outer diameter = 0.72 mm, Nordson EFD, USA). Medium flow was controlled by programmable pressure regulators (LineUP FlowEZ, FLUIGENT, France), which generated flow by applying pressure on 50-ml medium reservoirs (P-CAP series, FLUIGENT, France). Fresh and conditioned TB medium were respectively filled in separate reservoirs, and each one was pressurized by one module of the pressure regulator. After the cell inoculation both media were connected to the inlets of the channel via tygon tubing and blunt dispensing needles. The pressure at the inlet of the fresh TB medium was set to 200 mbar and remained constant throughout the experiment. During the selection of the positions for imaging the pressure at the inlet of the conditioned medium was set to 250 mbar, allowing the conditioned medium to flow through the junction to the mother machine growth sites and thereby maintaining the stationary state of the cells. At the beginning of imaging, the pressure at the inlet of the conditioned medium was reduced to 150 mbar and programmed to increase back to 250 mbar after 4 h of on-chip cultivation, thereby activating a medium switch from fresh to conditioned TB medium. Phase contrast and GFP fluorescence images were acquired with a time interval of 10 min.
