# Supplementary material for: Regulation by cyclic di-GMP attenuates dynamics and enhances robustness of bimodal curli gene activation in Escherichia coli
Source: PLoS Genet. 2023 May 15;19(5):e1010750. doi: 10.1371/journal.pgen.1010750 (PMC10212085; doi:10.1371/journal.pgen.1010750)
Supplement: S3 Protocol — (PDF) [file pgen.1010750.s014.pdf]

S3 Protocol. Analysis of the microfluidics data.

Cells were segmented from phase contrast images by making use of a fully convolutional neural network based on the U-net architecture. A set of manually curated cell outlines was prepared for training (1105 outlines) and validation (346 outlines) of the network. The training set was augmented by scaling, rotation, flipping and addition of white noise. A U-net of depth three, with 8, 16 and 32 filters along the contracting path, was trained to predict cell interiors from phase contrast images. Phase contrast images were normalized by subtracting the median and scaling to intensities expected between the 2nd and 98th percentiles. Cell interiors were defined from the curated outlines by filling each outline and then subjecting it to two rounds of morphological erosion. The erosion step ensured that neighbouring cells predicted by the network were well separated, such that distinct cell instances could be clearly identified simply by thresholding the prediction and labelling connected regions. After instance identification, two rounds of morphological dilation restored each mask to its original size. Finally, a smooth outline for each cell was obtained as a two-dimensional spline defined by equidistant knots placed on the mask edge.

To track cells between time points, we applied a length conservation strategy for the cells along each trench. At each time point, we ordered cell outlines by their depth in the trench, with deepest cells first. We then attempted to match, in order, a cell outline in time point *t* with one or more cell outlines in time point *t*+1, chosen such that the sum of their cell lengths would be conserved within some threshold tolerance. In the trivial case, the length of the first outline at time point *t* would match that of the first outline at time point *t*+1. In the event of cell division, the cell length at time point *t* would match the sum of the first two cell lengths at time point *t*+1. Since cells may grow in length between time points, we also initialised a growth rate parameter for each cell that biased the expected cell lengths for time point *t*+1 as a fold-increase in length. To enable adaptation to the true growth rate, the growth rate parameter was updated by a lagging average over 20 time points. To increase robustness to errors in segmentation, we additionally allowed state transitions from one to many and many to one, and built a proposal tree, which branched for all valid assignments lying within the length thresholds. We searched for the proposal with the lowest average fold-change in matched lengths, but limited branching by retaining only the 10 best proposals for subsequent nodes (cell outlines) in the tree. The length thresholds were deliberately set loosely such that the (sum of) cell length(s) at *t*+1 could decrease at most five-fold or increase at most two-fold relative to the (sum of) cell length(s) at *t*. This increased the number of valid proposals, but was important in cases where the growth rate estimate was poor. For transitions where one cell outline split into more than two, or transitions where multiple outlines merged into one cell, a new label was generated for the corresponding cells at *t*+1. We made one exception to this labelling strategy to account for occasional ambiguity in segmentation near division events, where a cell segmented as two

sister cells could later be segmented as a single mother cell. Specifically, when two sister cells — i.e., cells that were previously involved in a division event — merged into one, the label was set back to that of the mother; at the next division event, the labels of the sister cells were also retained. Finally, note that any outlines below a minimum size threshold of 50 pixels were ignored. All errors in tracking were manually curated.

Cell length was estimated from cell regions as the 'major axis length' of the Matlab regionprops function — the major axis of the ellipse with same normalised second central moment as the region. Instantaneous growth rates were estimated from the derivative of a smoothing spline fitted to the logarithm of cell length over each cell division cycle. Knots for the spline were placed at intervals of at most 15 time points. Single-cell fluorescence traces were quantified from the median fluorescence within each outline. Background fluorescence varied as a function of time due to the accumulation of cells at some trench exits, so we corrected for background fluorescence in each trench at each time point using the median value of all non-cell pixels. Fluorescence traces were characterised along branching lineages and were smoothed with a Savitzky-Golay filter of order 3 and window length 21. The derivative of the filter was used to obtain the maximum rate of fluorescence increase. Peaks in fluorescence were identified as points for which the second derivative was less than -100 and either the first derivative was zero, or its sign changed at the next time point. In cases where multiple descendants shared a common peak event before branching, we counted that event only once. The half-life of decay after a peak in fluorescence was determined by fitting an exponential decay function with offset term to fluorescence data after the peak. Given the occasional occurrence of a second peak in fluorescence, the decay function was only fit to time points after the peak up to the point where the first derivative exceeded zero.
