# Supplementary material for: Regulation by cyclic di-GMP attenuates dynamics and enhances robustness of bimodal curli gene activation in Escherichia coli
Source: PLoS Genet. 2023 May 15;19(5):e1010750. doi: 10.1371/journal.pgen.1010750 (PMC10212085; doi:10.1371/journal.pgen.1010750)
Supplement: Attachment — Submitted filename: Response reviewers resubmission.docx [file pgen.1010750.s019.docx]

**Point-by-point responses to Reviewers**

Reviewer #1: The manuscript PGENETICS-D-22-00592 by Lamprecht et al. aims to reveal the regulation of curli activation by c-di-GMP via monitoring curli expression in individual E. coli cells using flow cytometry and a self-designed microfluidic device. The study is interesting and the paper is generally well-written and accessible.

*We would like to thank the Reviewer for the overall positive feedback on our study and for the valuable comments.*

However, the following points should be considered by the authors:

1. Figure 1A and Figure S1A: Y-axis of the growth curves should be OD600, but not log10OD600.

*We apologize for this mistake and thank the Reviewer for pointing it out. The labels are now corrected.*

2. Some supplementary figures should be provided as figures to increase the readability of the paper. Such as Figure S8, Figure S11, Figure S13.

*We have now moved Figure S8 to the main text as a new Figure 5, and added Figures S11 and S13 as new panels to Figure 6 (previously Figure 4) for better readability, as the Reviewer suggested.*

3. Figure S7: Flow cytometry measurements of curli expression in macrocolony biofilms. When the cells in macrocolony were suspended in liquid, their state has been changed. Thus, the authors should give controls to make sure that flow cytometry measurements of curli expression can represent curli expression in macrocolony biofilms.

*Although the environment and therefore the physiological state of cells indeed changes when the microcolony biofilm is resuspended in the liquid, cells are sorted immediately after this resuspension, as described in Methods. Since the levels of (stable) GFP reporter used in our experiments are not expected to change in absence of cell growth, we believe that they provide a faithful representation of the reporter activity in the microcolony biofilm before harvesting. We now comment on this in the text.*

4. Figure 2A: Regulation of curli gene expression by c-di-GMP in *E. coli* are attributed to two DGCs and two PDEs. If E. coli encodes other DGCs and PDEs, it is better to include a control deleting a gene encoding another DGC or PDE in the flow cytometry measurements.

*In this study we focused on the role of the established c-di-GMP regulatory network of curli expression in E. coli (shown in Figure 2A), including an upstream regulatory module (DgcE/PdeH) that controls global levels of c-di-GMP and the local downstream module (DgcM/PdeR) that controls activity of the regulator MlrA. Our results demonstrated that this network is dispensable for the induction of bimodal curli expression. We further showed that removal of the local downstream module (DgcM/PdeR) makes curli regulation insensitive to the global level of c-di-GMP. This result was presented in Figure S3 of the original manuscript, but we now moved it to the main text as the new Figure 3, in order to highlight it more prominently.*

*E. coli indeed has more DGCs and PDEs, which could contribute to global levels of c-di-GMP and thereby curli expression in the wildtype cells (although DgcE and PdeH make the largest contributions). However, since curli expression becomes decoupled from such global changes in c-di-GMP once the local regulation by DgcM and PdeR is removed, these contributions should not affect curli gene expression.*

*What we indeed cannot exclude – although we find this unlikely – is a hypothetical regulation of curli expression by some local DGC/PDE module that is itself not sensitive to the global c-di-GMP levels.*

*We have accordingly refined the text to emphasize that our analysis addresses global and local c-di-GMP regulation of curli by the established DGC/PDE modules, but it does not rule out involvement of other hypothetical (insulated) local DGC/PDE modules.*

Reviewer #2: In this work, the authors investigate the bimodal expression of curli fimbriae in an *Escherichia coli* K-12 laboratory strain using a transcriptional reporter downstream of the *csgA* gene of the *csgBAC* operon encoding the major subunit of curli. They show that in three different biofilm models, cell aggregation in liquid culture, in a flow cell device and upon macrocolony formation on agar plates the role of two hierarchically organized diguanylate cyclase/phosphodiesterase pairs regulating the turnover of the second messenger cyclic di-GMP dedicated to expression of the biofilm regulator csgD regulate curli expression distinctly in the different models.

*We apologize to this Reviewer for being apparently unclear in the description and interpretation of our results. Our data actually show that the regulation of curli by c-di-GMP, as mediated by the two DGC/PDE pairs, is qualitatively similar in all tested model systems including microfluidics. In all cases, the deletions of DGC genes consistently lower the number of curli-positive cells and the deletions of PDE genes increase it. These control data (for liquid cultures and biofilms) have been presented in Figure 2 and Figures S4-S7 in the previous version of our manuscript. We have now moved these controls for individual gene deletions to the main text to improve clarity. Furthermore, we have performed additional control experiments in the microfluidic device to confirm that individual deletions also behave consistently in this case.*

*We also show that, in all of these models, the activation of curli expression is bimodal, and this bimodality does not require the two studied DGC/PDE pairs and in their absence no longer depends on the global level of c-di-GMP (also see our response to #4 of Reviewer 1).*

*The primary difference between each of the biofilm models is the fraction of curli-positive cells, which is moderately varying in the wildtype but much more strongly in the strains lacking both (or only the lower) DGC/PDE pairs. This observation – along with faster but more variable switching of curli reporter expression in the absence of established regulation by DGC/PDE pairs in the microfluidics device – led us to conclude that c-di-GMP dependent regulation is not the cause of bimodal curli gene expression but enhances its robustness. These key conclusions are now discussed clearer in the text.*

General comments

    While the authors have confirmed that regulation of csgD (indirectly through their monitoring of curli fimbriae) is dependent on two functionally hierarchically arranged diguanylate cyclase/phosphodiesterase pairs in liquid medium, they originally show that this is not the case in other biofilm model system. Their conclusion, however, that the second messenger cyclic di-GMP is not involved in the expression of curli in these model system is, based on the experimental data presented, not justified at this point. The authors need to show that curli expression is not regulated by alternative diguanylate cyclases/phosphodiesterases under the different growth conditions. This can be done by overexpression of a potent diguanylate cyclase/another phosphodiesterase.

*As noted above and in our response to #4 of Reviewer 1, our data do show that curli expression is regulated by the upstream modules DgcE/PdeH and DgcM/PdeR in all of the tested models, and we revised our figures and text to make this point clearer. However, removal of these regulatory modules does not abolish bimodality of curli expression, which is again true in all tested growth models. We have also performed the exact experiment the Reviewer is asking for: by overexpressing DgcE or PdeH, which are respectively the most potent DGC and PDE in E. coli, we show that in the absence of the DgcM/PdeR module, the expression of the curli reporter is decoupled from the global level of c-di-GMP. We have now moved Figure S3, which showed this result, to the main text.*

In Salmonella typhimurium, for example, at least four diguanylate cyclases are involved in the expression of csgD grown as microcolonies and it has been shown that different cyclic di-GMP genes can be required under different growth conditions. They also need to couple the effect of the specific genes to the catalytic activity of the gene products in order to discriminate cyclic di-GMP production from potential scaffold effects. Third, it has been indicated in S. typhimurium (Kader et al., Mol Microbiol, 2006) and E. coli (Sommerfeldt et al, Microbiology, 2009; Tagliabue et al, FEMS Immunol Med Microbiol, 2010) that the curli genes are regulated beyond csgD expression by cyclic di-GMP/diguanylate cyclases under different growth conditions thus adding an additional level of regulation that has not been considered by the authors.

*We thank the Reviewer for raising this point. As discussed in our response to #4 of Reviewer 1, we indeed cannot rule out that some alternative local DGC/PDE module that is insensitive to global levels of c-di-GMP regulates curli expression independently of the DgcM/PdeR module. We have now modified the text accordingly, to mention this possibility.*

*Regarding the discrimination between catalytic activity and scaffolding effects, it is a valid question, particularly for the DgcM/PdeR pair where enzymatic and scaffolding effects are known to be intertwined. But since bimodal curli activation is still observed even in the absence of both regulatory pairs, we believe that making this distinction is not important for our manuscript.*

*We further thank the reviewer for drawing our attention to references reporting c-di-GMP effects of curli expression that are independent of csgD expression. These are now mentioned along with the previously cited reference (Ahmad et al).*

Specific comments

Abstract: Is missing the information about bimodal expression of curli in the microcolony.

*The abstract has been modified accordingly to mention all tested models of curli expression.*

l.20: planktonic cells include single cells and, per original definition, flocks or cell aggregates. However, with respect to biofilm formation, a stricter definition is required as cell aggregates, although present in the liquid culture, are considered as a specific type of biofilm that, at least in case of S. typhimurium (Gerstel and Römling, Environ Microbiol, 2001) is also regulated by csgD expressing curli.

*We fully agree that cell aggregates should be considered as a specific type of biofilm. However, under our growth conditions in liquid culture with strong shaking, we do not observe formation of such aggregates. We apologize for not explicitly mentioning this in the previous version of the manuscript; this has now been corrected and also the reference suggested by the Reviewer has now been cited. And our observation of comparable curli reporter induction in microfluidic channels, where no cell aggregation is possible, further confirms that curli gene activation does not require aggregation.*

l.25: based on the results presented in this manuscript, I do not agree with this conclusion by the authors.

*We modified this sentence to make it more specific, see discussion above.*

l. 150: Is this a stable or instable GFP? Was, at some point, a correlation between curli production and activity of the transcriptional fusion performed?

*Our transcriptional reporter harbors a stable GFP, which is now mentioned. In our previous publication where this reporter was first described (Besharova et al., 2016), we show that its activation correlates with curli-dependent biofilm formation and cell recruitment to aggregates within submerged biofilms. This is now mentioned in the text.*

l.185: knockout? deletion mutants

*The text has been modified throughout accordingly.*

l.207: The conclusion here can be that cyclic di-GMP produced by DgcE targets the csgD promoter and curli specifically through DgcM under these conditions. Were alternative strong diguanylate cyclases overexpressed to verify the conclusion of cyclic di-GMP independence?

*In this respect, our data are indeed consistent with the previous work from the Hengge lab and confirm that under all tested conditions the c-di-GMP regulatory network operates as shown in Figure 2A. But – in contrast to this previous work – we conclude that the bimodality of curli expression does not require this network. This is discussed even clearer now in the text. As also mentioned above in response to general comments by this Reviewer, we further show that global changes in the levels of c-di-GMP no longer affect curli gene expression when DgcM/PdeR module is absent. See further replies below, too.*

l.208: Figure S4: Is there a difference in transcription of the csgBAC operon between the wild type with or without vector control?

*We assume that the Reviewer refers to Figure S3 (which has been now remade and moved to the main text as Figure 3). Comparison of this figure with Figure 2B shows that the empty vector has little effect on the bimodality or levels of reporter expression in the overnight culture. We now mention this in the text.*

l.240:  Was it excluded that other cyclic di-GMP turnover proteins are involved in transcriptional regulation of curli under these growth conditions?

*As discussed above, we modified the text accordingly to state that we ruled out that the DgcE/PdeH and DgcM/PdeR modules are required for bimodality of curli expression (under all tested conditions) and that, in the absence of the DgcM/PdeR module, global levels of c-di-GMP have no effect. We would like to reemphasize here that all of the individual deletions of these DGC and PDE enzymes do affect curli expression. And as long as the DgcM/PdeR module is present (i.e., in the wildtype cells), other enzymes could also affect curli expression by changing global c-di-GMP levels.*

*What our experiments indeed cannot entirely rule out is that curli gene expression is additionally regulated by some hypothetical local c-di-GMP regulatory module that is insensitive to the global level of c-di-GMP. While we believe that such scenario is rather unlikely, we explicitly mention this possibility in the text.*

l.265: While cyclic di-GMP independent regulation of curli (through csgD?) might be possible, this data does not show this convincingly. It might just be that expression of curli is ‘blind’ to the regulation of DgcE/DgcM/PdeH/PdeR under these conditions.

*Again, we never concluded that curli regulation is “blind” to these enzymes under any of the tested conditions, and the effects of deletions of individual enzymes are fully consistent with previously published data. Nonetheless, we do see that curli activation occurs and remains bimodal even when both modules are removed. However, in our microfluidics data to which this line refers, this activation happens faster and in more heterogeneous fashion.*

l.282: Reference 29 and earlier work in S. typhimurium do not state positive feedback loop of csgD expression. This was shown for E. coli. Instead it shows that bimodality of csgD expression is globally regulated by cyclic di-GMP signaling.

*We apologize for not referring to the Salmonella work correctly. We have now modified the text accordingly.*

l.291: Nitrogen and phosphate depletion have also been shown to stimulate the csgD promoter. Were the stimulatory effects of conditioned medium with respect to depletion of individual amino acids specifically dissected?

*Our results show that curli expression is suppressed upon addition of serine (which is known to be the first amino acid to be depleted from E. coli culture medium under these conditions) and conversely induced by addition of its non-metabolizable analogue SHX. We therefore believe that depletion of amino acids is a likely inducer, but we have not specifically tested depletion of other medium components. We rephrased the text accordingly.*

Figure 1: In this type of experiment, it might be worthwhile to try to avoid the effect of reporter protein stability upon inoculation at the beginning of the growth curve (Gerstel and Römling, Environ Microbiol, 2001).

*Although we do agree that the level of curli reporter at inoculation (Figure 1A and other figures showing plate reader data) could be reduced by using cells from an early exponential growth phase as inoculum, we do not think it is necessary: as seen in Figure 1A, essentially all cells in the culture first turn off curli expression in the course of growth, before turning it on again. But we thank the Reviewer for pointing this out and we specifically comment on the initially high level of culri expression when describing this experiment in the Results.*

Reviewer #3: The manuscript by Lamprecht et al. analyzes the bimodal activation of curli expression in E. coli planktonic cultures. Results confirm previously published results showing bimodality of curli expression under planktonic incubating conditions. Through the analysis of bimodality shown by several mutants in the circuitry that dictates MlrA activity and thus, activation of csgD transcription, authors lead to the conclusion that c-di-GMP is not required for curli gene activation and bimodality. Furthermore, the study addresses curli expression at the single cell level in a microfluidic device using a conditioned medium in order to mimic nutrient depletion conditions.

The paper is reasonably written and the results are shown in a straightforward manner.

*We thank the Reviewer for this summary of our results and for critical comments and suggestions on improving the manuscript. Regarding the summary, we’d like to emphasize that we do observe that up- or downregulation of c-di-GMP affects curli gene expression, largely consistent with the work of the Hengge group that we cite extensively. However, as absolutely correctly stated by the Reviewer, in contrast to this previous literature our data show that this regulation by c-di-GMP is – surprisingly – not required for activation or bimodality of culri gene expression.*

*Nevertheless, we report important quantitative differences in curli induction between the wildtype and the strain lacking c-di-GMP regulation, as shown in Figure 4 and Figure 6. We rephrased the text to emphasize these points more clearly.*

Major comments

Curli expression depends on the transcriptional regulator CsgD. Bimodality of csgD expression and thus curli production in a planktonic culture of E. coli during entry into stationary phase has already been nicely demonstrated by the group of Regina Hengge (Serra et al. J. Mol. Biol. 431, 4775–4793). Therefore, the first part of the present study shows no novelty and confirms previous results through the use of another reporter system (PcsgA::gfp transcriptional fusion instead of PcsgD::gfp reporter fusion used by Serra et al.)

*First of all, we cite this study multiple times (Ref. 27 in our original manuscript), both in the Introduction and in the Discussion section when referring to the bimodality of curli expression, along with earlier studies where similar bimodality (interpreted as bistability) was reported for Salmonella. However, there are a number of important differences between the bimodality of csgD expression reported in Ref. 27 and the bimodality of the csgA reporter expression observed in our work, with respect to the timing and degree of bimodality (which emerges earlier and is much more pronounced for the csgA reporter) as well as to the dependence of bimodality on the deletions of DGC and PDH genes. As mentioned already in the Discussion in the previous version of our manuscript (and now explained more extensively), these differences suggest that the bimodality of csgD expression is unlikely to be the (only) cause for the bimodality of csgA expression. In this respect, our results are certainly much more than confirmatory.*

On the other hand, conclusions about the role of c-di-GMP in bimodality of curli expression are confusing and based in misinterpretation of already published results.

*We strongly disagree. As mentioned above (as well as below), in many points our results are fully consistent with the previous results from the Hengge lab, and these relevant papers have been cited accordingly in our manuscript. However, in contrast to the conclusions drawn in this earlier work based on expression of csgD regulator, our data clearly shows that c-di-GMP regulation is not required for the bimodality of expression of csgBA curli genes, which is one of the central points of our manuscript.*

*We would like to further emphasize that all of our conclusions are drawn based on our own data, and not on (mis)interpretation of earlier results. We now elaborate more extensively on these similarities and differences between our results and the previously published work, in order to make it clear where we agree and where we disagree.*

1.  The MerR-like transcription factor MlrA interacts with the diguanylate cyclase DgcM, forming a complex that activates transcription of csgD. In still growing cells that experience low c-di-GMP levels due to high activity of the phosphodiesterase PdeH, the PdeR enzyme inhibits DgcM and MlrA by direct interaction and, as a consequence, csgD is not expressed. When c-di-GMP production by the σs-induced diguanylate cyclase DgcE increases, binding and cleavage of c-di-GMP by PdeR releases DgcM and MlrA, resulting in initiation of csgD transcription (Lindenberg et al. Embo J 32, 2001–2014). Thus, the PdeR/DgcM switch module is the one responsible for heterogeneity of csgD expression (Serra et al. J. Mol. Biol. 431, 4775–4793). Taking into account that the levels of c-di-GMP dictate PdeR binding capacity to DgcM, the general conclusion of the present study, that is “the upstream regulation by c-di-GMP is not required to establish the bimodality of curli expression” is not right.

*We absolutely agree with the first part of the Reviewer’s summary of the csgD regulation by DgcM and PdeR, which is exactly what is stated in our Introduction and shown in Figure 2A. Both the Lindenberg et al paper mentioned by the Reviewer and other papers from the Hengge group have been cited accordingly. But although the study by Serra et al indeed concluded that the PdeR/DgcM switch module is responsible for heterogeneity of csgD expression, our results clearly show that this is not the case for the bimodality of csgA expression downstream of csgD. As mentioned above, this prompted us to question the assumption of the Reviewer that the (moderate) heterogeneity of csgD expression observed by Serra et al is the (main) cause of csgA bimodality. Although MlrA and CsgD themselves are clearly required to activate csgA expression, these are not the only regulators contributing to csgA expression, and our data indicate that the heterogeneity of csgA expression originates elsewhere. This is now discussed more extensively in the revised version of the manuscript.*

In fact, most of the results shown in this manuscript (figure 2) support previous findings by Serra et al under planktonic conditions:

-mlrA mutant : 0% of csgA positive cells

-pdeH or pdeR mutant: almost 100% of csgA positive cells

-dgcE or dgcM mutant: very small fraction of csgA positive cells

*Again, we agree that effects of individual mutations are fully consistent with multiple previous papers from the Hengge lab, which are cited extensively.*

- a double pdeH dgcE mutant: 30% of positive cells. This can be explained as follows: a pdeH mutant shows a 10- to 15-fold higher c-di-GMP level than a wild type strain. A secondary deletion of dgcE lowers c-di-GMP, but still remains 3- to 4-fold higher in the double mutant than in the parental wild-type strain. Thus, lines 188-89 are not correct.

*We strongly disagree. The statement says that in our experiments (!) similar bimodality of curli expression is observed in this double mutant and in the wildtype cells. This is clearly the case, even though the fraction of positive cells is lower compared to the wildtype.*

- As regards the double pdeR dgcM mutant and the quadruple pdeH dgcE pdeR dgcM mutant, 30 and 43 % of cells show csgA activation, respectively. In this scenario MlrA would be active because of the absence of PdeR, though absence of DgcM might not allow full MlrA activity. Thus, again, conclusions summarized in lines 194-97 are not necessarily right.

*Again, the statement in our text refers to the pronounced bimodality of curli expression, which is retained in this double knockout. It is thus correct and supported by our data.*

Again, as regards lines 203-207, insensitivity of the quadruple mutant to c-di-GMP levels is totally expected, since it is already known that MlrA does not bind c-di-GMP, and in these mutant cells, MlrA activity is dictated by the absence of PdeR and DgcM.

*We principally agree, but we nevertheless believe that this experiment represents an important control (see related comments of the other two reviewers). Given the importance of this control, we have now moved these results to the main text.*

In order to test if, under the conditions used in this study, DgcM is not dispensable in the absence of PdeR and thus, for full MlrA activity, interaction with DgcM is needed, a triple pdeH dgcE pdeR mutant should be constructed and csgA expression and bimodality could be compared to that in the wild type and quadruple mutant strains.

*Since we observe the bimodality of csgA expression even in the absence of DgcM, it is clearly dispensable for bimodality. But we agree that the triple deletion strain suggested by the Reviewer is interesting to test the impact of DgcM in the absence of the other components. We have thus constructed this mutant and analyzed reporter expression in this background. The results are now included in Figure 2. Consistent with the previous work of the Hengge group (which we referred to already in the previous version of the manuscript (“…DgcM might also activate MlrA through direct protein interaction”*), these results suggest that *DgcM has indeed an activatory effect on curli expression even in absence of PdeR. We comment on this in the text. But with or without DgcM, the bimodaliy of expression is still observed, confirming that DgcM is dispensable for bimodality.*

2.  Figure 3, Fig S5: again, a triple pdeH dgcE pdeR mutant should be tested and csgA expression and bimodality should be compared to that in the wild type and mutant strains.

*See above. Since DgcM is clearly dispensable for bimodality, we only measured this triple deletion strain in liquid TB but not under other conditions.*

Differences in curli expression and bimodality found when E. coli is grown under distinctive conditions (plate reader, submerged biofilms, microcolony biofilms) may be due to differences in mlrA expression and/or csgD expression in different environmental conditions. In fact, it is known that high osmolarity represses transcription of csgD. The TB medium used in this study contains 5 g NaCl per liter, and thus, expression of csgD is expected to be lower than in the case of microcolony growth conditions (LB without salt). In order to compare conditions of culture growth, LB without salt should be used along the study. Thus, to my opinion, results summarized in lines 239-241 are misinterpreted.

*It is certainly possible that the differences in the fraction of curli-positive cells are indeed due to different expression levels of mlrA or csgD, but this is not relevant for our conclusion that the variation in the fraction of curli-positive cells from condition to condition is larger in the absence of c-di-GMP regulation than in the wildtype cells. Thus, our statement is fully supported by our data. Further, in our opinion, differences in osmolarity also count as differences in growth conditions. Nevertheless, we now performed an additional experiment to investigate the activation of csgA reporter in LB without salt as suggested, which is now shown in Figure 4. Although the exact expression pattern across deletion strains is somewhat different from both liquid TB and from macrocolony biofilms on LB without salt, the bimodality in the quadruple deletion strain is also observed under these conditions. Interestingly, we observed that in LB without salt the fraction of curli-positive cells is even higher in the quadruple deletion strain than in the wildtype, which further supports our argument that c-di-GMP signaling is important to make this fraction less variable among different conditions.*

In summary, the study fails to address how the findings relate to previous research in this area. Also, data do not provide enough evidence for the author´s conclusions and the study does not show enough novelty.

*We absolutely disagree with these statements, as discussed in length above. In order to make it even more clear, we modified the text to improve the clarity of presentation and to highlight the commonalities and differences between our findings and the work of the Hengge lab.*
