# Supplementary material for: Regulation by cyclic di-GMP attenuates dynamics and enhances robustness of bimodal curli gene activation in Escherichia coli
Source: PLoS Genet. 2023 May 15;19(5):e1010750. doi: 10.1371/journal.pgen.1010750 (PMC10212085; doi:10.1371/journal.pgen.1010750)
Supplement: Attachment — Submitted filename: Response reviewers revision.docx [file pgen.1010750.s020.docx]

**Point-by-point responses to Reviewers**

**Reviewer #1:**

This is the revised version of a manuscript previously submitted to PloS Genetics.

While the description of the work has significantly improved, I still have the following comments (not considering that some of the controls like assessment of catalytic mutants of cyclic di-GMP turnover proteins have not been performed in any case).

*We thank this Reviewer for acknowledging that the manuscript has significantly improved upon revision. Regarding the Reviewer’s comment about assessment of the catalytic mutants of DGCs or PDEs: Although such analysis might indeed be interesting for the follow-up work aiming to elucidate how exactly the curli gene expression is stabilized by the PdeR/DgcM module, we believe that it is not essential for our study and would go beyond the scope of this manuscript.*

First, to state that cyclic di-GMP does not substantially affect bimodality (bistability) is not necessarily justified as this work shows that deletion of individual PDEs in two instances shifts >99% (?) of cells to expression. Where is the threshold for definition of a bimodal/bistable expression (at a specific growth condition) and how do genes behave that are not bimodally expressed? Is there any reference for this?

*We have never stated that c-di-GMP does not substantially affect bimodality of curli gene expression. For example, in the concluding sentence of the Abstract we clearly say: “*We thus conclude that, while not required for the emergence of bimodal curli gene expression in E. coli, this c-di-GMP regulatory network attenuates the frequency and dynamics of gene activation and increases its robustness to cellular heterogeneity and environmental variation.*” Also the title of our manuscript clearly states that c-di-GMP has an important regulatory function. What we indeed observe is that this regulatory network is not required for the bimodality to emerge, which is most obvious and clear in the E. coli strain where this entire c-di-GMP network is missing. This strain nevertheless shows very clear differentiation into subpopulations of curli-positive and negative cells, which under most tested conditions have comparable size. Such differentiation is the hallmark of bistability/bimodality of gene expression, whereas monomodally expressed genes show only one peak in the distribution of their expression levels (that might be narrow or broad). This topic has been extensively investigated, both experimentally and theoretically, and as suggested by the Reviewer we have now added a brief discussion of general mechanisms that could lead to bimodality along with the references to the relevant literature in the Discussion section.*

The control experiment with overexpression of a diguanylate cyclase to demonstrate ‘blindness’ to cyclic di-GMP involves the same DGC as deleted in the regulatory pathway. Another DGC which shows potent DGC activity such as AdrA or a constitutively activated response regulator-DGC is necessary to make an initial conclusion. It has been demonstrated in screens (examples can be found in Kulasakara et al, PNAS, 2006) that despite of high cyclic di-GMP production, cells can be ‘blind’ to the observed physiological changes and vice versa.

*Although we find this scenario very unlikely, it is already explicitly mentioned several times in our manuscript that we cannot rule out involvement of some hypothetical local DGC/PDE module that is insensitive to the global c-di-GMP. For example, we state in the Discussion section: “*Since removal of the DgcM/PdeR module further made curli gene expression insensitive to global levels of c-di-GMP, contributions of other E. coli DGCs and PDEs to curli regulation in this deletion background are unlikely, even if these enzymes might in principle affect curli gene expression in wild-type E. coli cells via the common c-di-GMP pool. We therefore conclude that c-di-GMP regulation might be dispensable for activation or bimodality of curli gene expression in E. coli, although we could not rule out regulation of curli gene expression by a hypothetical local DGC/PDE module that is insensitive to global c-di-GMP levels.*”*

*Moreover, following the suggestions of the Reviewer #2 we have now revised the manuscript to emphasize even more clearly that our study specifically addresses the role of the two established DGC/PDE regulatory modules, DgcE/PdeH and DgcM/PdeR. E.g., the second subheadings in the Results section states* “Bimodality of curli gene expression does not require the c-di-GMP regulatory network composed of DgcE/PdeH and DgcM/PdeR pairs”. *Finally, the Abstract has been also modified accordingly.* *We believe that such phrasing of our conclusions to specifically refer to the DgcE/PdeH and DgcM/PdeR pairs makes the additional control with overexpression of another unrelated DGC suggested by the Reviewer not necessary.*

*Concerning the specific expression of AdrA (=DgcC) suggested by the Reviewer, our preliminary unpublished experiments suggested* that *DgcC* has an indirect *impact on curli gene expression that could not be explained by changes in global c-di-GMP levels (it reduced curli expression in wild-type cells instead of increasing it, as would be expected from its DGC activity). Given the complexity of curli gene regulation, the overexpression of other DGCs might have similarly pleiotropic effects. We are currently working on a systematic and thorough characterization of effects of all E. coli DGCs and PDEs on curli gene expression, but this is a separate ongoing project. However, as mentioned above, the modifications made to the revised text to emphasize that our conclusions specifically relate to the DgcE/PdeH and DgcM/PdeR pairs already address this Reviewer’s concern.*

Despite the rigorous conclusions drawn and, admittedly sophisticated experimental methodology, the initial theoretical design is rather sloppy as the MlrA, PdeH/DgcE and PdeR/DgcM regulatory network has been shown for csgD expression while expression of the csgBA operon is monitored. To the knowledge of this reviewer it has not even been formally demonstrated whether mlrA is involved in transcription of the csgBA operon. My suspicion would be that it is not.

*We thank the Reviewer for appreciating the rigor and sophistication of our study. We have never claimed or assumed that MlrA directly regulates the expression of csgBA operon. We believe that Figure 2A makes it clear that we consider the regulatory scheme (adapted from Sierra & Hengge, 2019) that MlrA regulates csgD, and CsgD then activates csgBA expression. Although this was already stated in the text of the previous version of our manuscript, we have now made it now even clearer in the revised version, by replacing “curli expression” with “csgBAC expression” in Figure 2A, and also by specifically stating that “*CsgD controls expression of the *csgBAC* operon that encodes the major curli subunit CsgA” in the legend of Figure 2A.

*The figure and the legend have now been corrected accordingly.*

References end at reference 47, but more are cited in the text.

*We apologize for this mistake; it is now corrected.*

Curli only expression leads to cell aggregation, but these interactions can be easily disrupted.

*We agree with this point, which is the advantage of using this strain for the analysis of the planktonic culture. We now mention it specifically in the text.*

l. 186: do you mean: …formation of suspended biofilm-like aggregates is not correlated with curli gene induction…

*We mean that it is not the consequence of aggregation. The sentence has been rephrased to improve clarity.*

l. 201: which percentage?

*The percentages were previously shown in Figure 2 but we now additionally specified them in the text.*

l.223: …in the cell population.

*Modified accordingly.*

l.313: In the movie S2, in a pdeH deletion mutant less csgA expression seem to be observed. Why?

*This is similar to difference observed between different batch growth conditions, where absolute frequencies are not the same as in the flask cultures. We now explain it better in the text.*

Reviewer #2: In this revised manuscript, Lamprecht et al. analyze the bimodal activation of curli expression using a transcriptional reporter for the csgBAC operon and flow cytometry. A main finding is described: bimodality of curli expression does not require the upstream modules formed by the DGC/PDE pairs DgcE/PdeH and DgcM/PdeR, although this network attenuates the frequency and dynamics of gene activation and increases its robustness to changing environmental conditions. I thank the authors for their extensive revision of the work, modification of the text and additional controls. Most of my previous concerns were addressed adequately. However, there are still minor points that remain to be addressed.

*We thank the Reviewer for appreciating the extent of our manuscript revisions. We have addressed all remaining points, as explained below.*

1. Abstract: line 30: the study can not rule out the involvement of other DGCs and thus c-di-GMP synthesized by them, in regulation of csgBAC expression. I suggest to change the sentence to “.. .shows stronger dependence on growth conditions in the absence of DgcE/PdeH and DgcM/PdeR regulation.”

*The sentence has now been modified to “…in the absence of regulation by DgcE/PdeH and DgcM/PdeR pairs”.*

2. Line 193: For more clarity, I suggest to change the title of this section to: Bimodality of curli gene expression does not require the c-di-GMP regulatory network comprised of the DGC/PDE pairs DgcE/PdeH and DgcM/PdeR.

*We modified the sentence accordingly.*

3. Line 218: Change culri to curli

*We apologize for the mistake; it’s now corrected.*

4. Line 224: Change: of the level…. To “on the level”.

*Thank you; it’s now corrected.*

5. All along the study, results indicate that in the absence of the regulatory network composed of DgcE/PdeH and DgcM/PdeR, MlrA is free to activate csgD transcription, and thus csgBAC expression, although is activity is lower than in the wild type strain because of the absence of DgcM. Since MlrA does not bind c-di-GMP, global changes in c-di-GMP do not impact curli expression or bimodality in the absence of DgcM/PdeR (Fig 3). Therefore, bimodality in the absence of this network seems to be caused by bimodality in mlrA expression. I understand that analyzing the biomodality of mlrA expression is out of the scope of this manuscript, but this issue should at least be discussed along the article.

*We believe that it might be a combination of mlrA, csgD and csgBAC regulation that is required for bimodality, which is now mentioned in the discussion. Elucidating the exact mechanism of bimodality will be subject of our future work.*

6. Also, although curli expression may be regulated by c-di-GMP independently of csgD transcription, the study shows that a mlrA mutant is completely negative for curli expression and that bimodality disappears. This also should be discussed.

*See our response above. We added a sentence commenting on the likely involvement of mlrA regulation in the emergence of bimodality. It is important to mention that at this stage we cannot rule out the involvement of other factors, including CsgD that is regulated by MlrA but also other TFs that regulate expression of MlrA, CsgD or of csgBAC operon itself.*

7. Line 283: Change “regulation by c-di-GMP” to “regulation by the regulatory network composed of DgcE/PdeH and DgcM/PdeR”

*The sentence was changed accordingly.*

8. Line 284: again, results could be due to a reduced bimodality of mlrA expression under microcolony biofilm forming conditions.

*We agree and now mention mlrA regulation in this context, although we cannot rule out other possible explanations.*
